# Using Google Glass in Nonsurgical Medical Settings: Systematic Review

**DOI:** 10.2196/mhealth.8671

**Published:** 2017-10-19

**Authors:** Bryn Dougherty, Sherif M Badawy

**Affiliations:** ^1^ Northwestern University Weinberg College of Arts and Sciences Evanston, IL United States; ^2^ Division of Hematology, Oncology and Stem Cell Transplantation Ann & Robert H. Lurie Children's Hospital of Chicago Chicago, IL United States; ^3^ Department of Pediatrics Northwestern University Feinberg School of Medicine Chicago, IL United States; ^4^ Department of Pediatrics, Division of Hematology and Oncology Zagazig University Faculty of Medicine Zagazig Egypt

**Keywords:** Google Glass, wearable, wearable device, head-mounted wearable device, non-surgical setting, non-surgical condition, medical setting, medical condition

## Abstract

**Background:**

Wearable technologies provide users hands-free access to computer functions and are becoming increasingly popular on both the consumer market and in various industries. The medical industry has pioneered research and implementation of head-mounted wearable devices, such as Google Glass. Most of this research has focused on surgical interventions; however, other medical fields have begun to explore the potential of this technology to support both patients and clinicians.

**Objective:**

Our aim was to systematically evaluate the feasibility, usability, and acceptability of using Google Glass in nonsurgical medical settings and to determine the benefits, limitations, and future directions of its application.

**Methods:**

This review covers literature published between January 2013 and May 2017. Searches included PubMed MEDLINE, Embase, INSPEC (Ebsco), Cochrane Central Register of Controlled Trials (CENTRAL), IEEE Explore, Web of Science, Scopus, and Compendex. The search strategy sought all articles on Google Glass. Two reviewers independently screened titles and abstracts, assessed full-text articles, and extracted data from articles that met all predefined criteria. Any disagreements were resolved by discussion or consultation by the senior author. Included studies were original research articles that evaluated the feasibility, usability, or acceptability of Google Glass in nonsurgical medical settings. The preferred reporting results of systematic reviews and meta-analyses (PRISMA) guidelines were followed for reporting of results.

**Results:**

Of the 852 records examined, 51 met all predefined criteria, including patient-centered (n=21) and clinician-centered studies (n=30). Patient-centered studies explored the utility of Google Glass in supporting patients with motor impairments (n=8), visual impairments (n=5), developmental and psychiatric disorders (n=2), weight management concerns (n=3), allergies (n=1), or other health concerns (n=2). Clinician-centered studies explored the utility of Google Glass in student training (n=9), disaster relief (n=4), diagnostics (n=2), nursing (n=1), autopsy and postmortem examination (n=1), wound care (n=1), behavioral sciences (n=1), and various medical subspecialties, including, cardiology (n=3), radiology (n=3), neurology (n=1), anesthesiology (n=1), pulmonology (n=1), toxicology (n=1), and dermatology (n=1). Most of the studies were conducted in the United States (40/51, 78%), did not report specific age information for participants (38/51, 75%), had sample size <30 participants (29/51, 57%), and were pilot or feasibility studies (31/51, 61%). Most patient-centered studies (19/21, 90%) demonstrated feasibility with high satisfaction and acceptability among participants, despite a few technical challenges with the device. A number of clinician-centered studies (11/30, 37%) reported low to moderate satisfaction among participants, with the most promising results being in the area of student training. Studies varied in sample size, approach for implementation of Google Glass, and outcomes assessment.

**Conclusions:**

The use of Google Glass in nonsurgical medical settings varied. More promising results regarding the feasibility, usability, and acceptability of using Google Glass were seen in patient-centered studies and student training settings. Further research evaluating the efficacy and cost-effectiveness of Google Glass as an intervention to improve important clinical outcomes is warranted.

## Introduction

Wearable technology is defined as any compact device, either in the form of a body sensor or head-mounted display, which provides a user information and allows user interaction via voice command or physical input [1]. The purpose of these devices is to create convenient, portable, and hands-free access to computers, thus facilitating or enhancing everyday tasks. Many of these devices can perform the same functions as mobile phones and laptop computers, while also outperforming them with their sensory and scanning abilities [2]. Google Glass (Google, Inc.), often referred to as “Glass,” which resembles standard eyeglasses, is one of the more well-known devices in this emerging field since its release in 2013 [3].

Google Glass has distinguished itself from other head-mounted or heads-up wearable devices by providing users with a comfortable, unobtrusive, wireless platform that runs the Android operating system and displays virtual or augmented reality with little obstruction to normal vision [3]. While it has not yet seen much success in the consumer market, various industries have taken an interest in the potential applications of a head-mounted, ubiquitous computer that could be used for a range of tasks, including recording and streaming videos, data transmission, telementoring in education, and teleconferences for professional collaboration [3]. Health care is one such industry that has pioneered research investigating how Google Glass could be leveraged to support both clinicians and patients.

Surgeons were among the first in the medical industry to incorporate Google Glass into their work. As a hands-free device that can react to voice commands, eye movements, and simple gestures, it is particularly attractive in environments where both hands are generally occupied with surgical tasks and maintaining sterility is of upmost importance [4]. In a recent systematic review, Davis and Rosenfield reported an overall positive impact of using Google Glass in surgical settings with data to support the feasibility and acceptability of its use for medical care, surgical skills training, medical documentation, and patient safety [4]. Many other specialties in medicine have followed the lead of the surgical field and conducted their own studies to assess the feasibility of using Google Glass in nonsurgical medical settings.

While Google Glass is an exciting technology with a number of promising applications in medicine, it remains unclear which applications are most worth pursuing, what potential limitations are associated with its use, and the extent to which patients and clinicians might benefit from its use. The objectives of this review are to systematically evaluate the most recent evidence for the feasibility, usability, and acceptability of using Google Glass in nonsurgical settings, and determine its potential benefits, limitations, and future directions in these settings.

## Methods

We followed the guidelines for the Preferred Reporting Items for Systematic Reviews and Meta-Analyses (PRISMA) in the reporting of evidence across the studies we reviewed (Multimedia Appendix 1) [5].

### Article Retrieval

A librarian collaboratively developed the search strategies with the senior author (SB) and ran searches in the following databases in November 2015: PubMed MEDLINE, Embase, INSPEC (Ebsco), Cochrane Central Register of Controlled Trials (CENTRAL) on the Wiley platform, IEEE Explore, Web of Science, Scopus, and Compendex. An updated search of all databases was run in January 2017 to look for additional articles. Search strategies for all databases except MEDLINE were adapted from the PubMed MEDLINE strategy. All databases were searched back to 2013, when Google Glass was first released. No language limits were applied. The search strategy specified keywords related to Google Glass. We also reviewed the search strategies of previous studies to include additional terms. See Multimedia Appendix 2 for complete search strategies in each database. An additional hand-search of related themes in the *Journal of Medical Internet Research* was also conducted. We also attempted to identify additional studies by searching the reference lists of key studies and relevant systematic reviews.

### Study Selection

The inclusion criteria were as follows: (1) original research articles, (2) studies that were either randomized controlled trials, quasi-experimental studies, or pilot/feasibility studies (including single arm, pre-posttest), (3) Google Glass interventions, (4) nonsurgical study settings, and (5) clinical, usability, feasibility, and/or acceptability as primary or secondary outcome. The exclusion criteria included (1) technology-based interventions other than Google Glass, (2) surgical study settings, and (3) articles with more technical description of Google Glass but no clinical, usability, feasibility, and/or acceptability outcomes.

### Data Extraction and Analysis

We used a standardized form for data extraction. Data items in the extraction form included the following: first author’s name, publication year, country, condition or disease focus of the study, purpose of the study, description of how Google Glass was used in the study as an intervention, participants’ age (when available), study design, study setting, duration of the study, and other study considerations. Two authors coded all included articles individually. Disagreements were resolved by discussion or by consultation with the senior author (SB), if needed. Quantitative and qualitative data analyses were conducted.

## Results

### Literature Search

**Figure 1:**
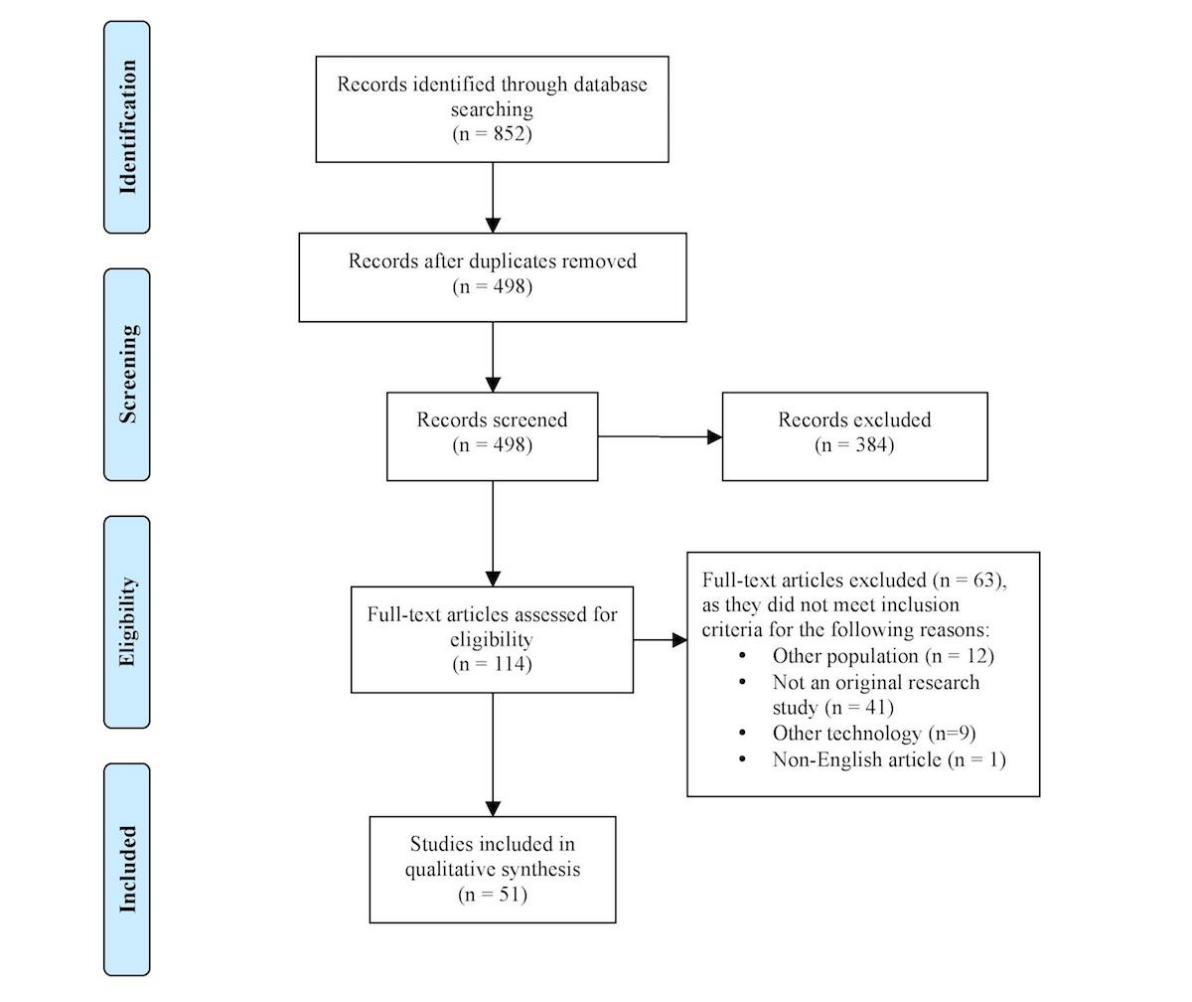
Flow of studies according to PRISMA guidelines.

The literature search identified 852 references (Figure 1), and 498 individual full articles were retrieved. A total of 51 articles met all inclusion criteria. Some of the interventions (21/51, 41%) studied the potential of Google Glass in aiding patients with a variety of conditions [6-26], while the majority (30/51, 59%) studied its potential uses in assisting health care professionals in their work [27-56]. The patient-focused studies aimed to help individuals with motor impairments (8/21, 38%) [6-13], visual impairments (5/21, 24%) [14-18], developmental and psychiatric disorders (2/21, 9%) [19,20], weight management concerns (3/21, 14%) [21-23], allergies (1/21, 5%) [26], or other health concerns leading them to track specific physiological metrics (2/21, 10%) [24,25]. The clinician-focused studies analyzed Google Glass use in student training (9/30, 30%) [35,37,38,40,41,46,48,49,52], disaster relief (4/30, 13%) [27-30], diagnostics (2/30, 7%) [32,50], nursing (1/30, 3%) [33], autopsy and postmortem examination (1/30, 3%) [53], wound care (1/30, 3%) [54], behavioral sciences (1/30, 3%) [31], and various medical specialties, including cardiology (3/30, 10%) [43-45], radiology (3/30, 10%) [39,42,47], neurology (1/30, 3%) [34], anesthesiology (1/30, 3%) [36], pulmonology (1/30, 3%) [51], toxicology (1/30, 3%) [55], and dermatology (1/30, 3%) [56].

### Description of Included Studies

Tables 1 and 2 summarize the characteristics of patient- and clinician-centered studies, respectively. In total, 40 studies were conducted in the United States [6-9,13-18,20-24,27,29,31, 32,35,37-41,43-52,54-57], three in Germany [25,26,53], two in United Kingdom [10,11], China [34,36], and one each in Australia [33], Switzerland [42], Mexico [19], Netherlands [12], Norway [30], and Italy [28]. Less than half of the included studies (19/51, 37%) were conducted in a laboratory setting [6-9,12,14,16-19,21-24,27,31,33,42,50], 13 (25%) in a hospital setting [32,34,36,38,39,43,44,46,51,53-56], seven (14%) in a classroom or clinical student training setting [35,37, 40,41,47,48,52], three in patient residences (6%) [13,15,20], three in local settings (6%) [28-30], and one in a dental office (2%) [49]. The remaining five studies were conducted in varying locations (10%) [10,11,25,26,45]. There was significant variability in information reported about participant demographics. Most (n=38) did not report any specific age information for participants [7,8,13-15,19-21,26-52,54-56], but none of these were conducted in pediatric settings. Of the 13 studies that did report participant age information, seven enrolled young adults (average age or age range ≤35 years) [6,18,22-25,53], three enrolled adults (average age or age range >36 and <60 years) [9,16,17], two enrolled older adults (average age or age range ≥60 years) [10,12] and one study reported an age range of 46-70 years [11]. Sample size ranged from 1-106 participants, with a median of 12 and a mean of 22 participants per study; 29 enrolled <30 [6,7,9-14,16-21,23-27, 30,38-40,43-45,48,53,54] and 10 had ≥30 participants [22,31,33,36,37,41,47,49,52,56]. Some of the studies (12/51, 23.5%) did not report the number of participants [8,15,28,29,32,34,35,42,46,50,51,55]. None of the studies reported information about participants’ race and ethnicity. Most (31/51, 61%) were pilot or feasibility studies [6-16,18,20-22, 24,26-29,32,33,35,36,38,47,48,53-56], six were randomized controlled trials (6/51, 12%) [30,31,37,40,41,52], five were exploratory studies (5/51, 10%) [42,44,45,49,50], five were case studies (5/51, 10%) [17,23,34,46,51], and four were quasi-experimental (4/51, 8%) [19,25,39,43]. None of the studies included any follow-up with participants after completion of the intervention.

**Table 1:** Summary of studies using Google Glass as patient-centered interventions.

Source (country)	Health condition	Study design	Study setting	Google Glass (GG) use
Anam et al, 2014 (United States)	Ophthalmology – visual impairment	Pilot/feasibility study	Laboratory	Monitors and reports nonverbal social cues to user
Garcia and Nahapetian, 2015 (United States)	Ophthalmology – visual impairment	Pilot/feasibility study	Patient home	Analyzes environment and reports the information to user to help them navigate a room
Pundlik et al, 2016 (United States)	Ophthalmology – visual impairment	Pilot/feasibility study	Laboratory	Magnifies user’s vision while completing a series of tasks
Hwang and Peli, 2016 (United States)	Ophthalmology – advanced age-related macular degeneration	Case study	Laboratory	Warps the vision of participants in efforts to improve vision
Tanuwidjaja et al, 2014 (United States)	Ophthalmology – colorblindness	Pilot/feasibility study	Laboratory	Helps participants identify colors
Lazewatsky et al, 2014 (United States)	Motor impairment	Pilot/feasibility study	Laboratory	Helps participants guide the robot personal assistant
Gips et al, 2015 (United States)	Motor impairment	Pilot/feasibility study	Laboratory	Allows people to operate a computer with only eye or head movements
Sinyukov et al, 2016 (United States)	Motor impairment – Locked-In Syndrome	Pilot/feasibility study	Laboratory	Uses voice control function of GG to allow people to navigate an electric wheelchair in indoor environments
Malu and Findlater, 2015 (United States)	Motor impairment – upper body impairment	Pilot/feasibility study	Laboratory	Uses touchpad and visual display to perform tasks on a computer/ mobile phone
McNaney et al, 2014 (United Kingdom)	Motor impairment – Parkinson’s Disease	Pilot/feasibility study	Varying locations (patient home, in public)	Helps in daily interactions and common activities
McNaney et al, 2015 (United Kingdom)	Motor impairment – Parkinson’s Disease	Pilot/feasibility study	Varying locations (patient home, in public)	Monitors user’s speech volume and provides feedback
Zhao et al, 2016 (Netherlands)	Motor impairment – Parkinson’s Disease	Pilot/feasibility study	Laboratory	Provides visual and auditory cues to modulate gait
Pervaiz and Patel, 2014 (United States)	Motor impairment – Dysarthria	Pilot/feasibility study	Assisted living facility	Helps people be aware of their volume, notifies them when to raise it, and provides feedback to clinicians so they can adjust therapy
Miranda et al, 2014 (Mexico)	Psychiatric/Developmental – Social Anxiety Disorder (SAD)	Quasi-experimental	Laboratory	Monitors symptoms of SAD through blinking habits
Voss et al, 2016 (United States)	Psychiatric/Developmental – Children with Autism Spectrum Disorder (ASD)	Pilot/feasibility study	Patient home	Uses the video feature to monitor everyday life
Mirtchouk et al, 2016 (United States)	Eating monitoring	Pilot/feasibility study	Laboratory	Records head motion while participants eat
Rahman et al, 2015 (United States)	Eating monitoring	Pilot/feasibility study	Laboratory	Records user’s eating and drinking habits through head movements
Ye et al, 2015 (United States)	Eating monitoring	Case study	Laboratory	Records head motion while participants eat
Hernandez et al, 2014 (United States)	Physiological measurements	Pilot/feasibility study	Laboratory	The accelerometer, gyroscope, and camera on GG are used to analyze the heart and respiration rate of user wearing the device
Richer et al, 2015 (Germany)	Physiological measurements	Quasi-experimental study	Varying locations (patients’ everyday lives)	Serves as the “wearable extension” portion of the DailyHeart app
Wiesner et al, 2015 (Germany)	Allergies	Pilot/feasibility study	Varying locations (drugstores selling cosmetic products)	Cross checks ingredients on cosmetic product package with a list of allergens created by the user in their online profile

### Description of Google Glass Use as Patient-Centered Interventions

Table 3 summarizes the Google Glass approach as patient-centered interventions. Five of the studies (5/21, 24%) used Google Glass to assist individuals with visual impairments or in low vision environments by providing them information about nonverbal social cues [14], allowing them to better navigate environments with the use of floor plans [15], improving vision magnification using mobile phone zoom capabilities [16], compensating for age-related vision impairments [17], and augmenting color perception [18]. Eight studies (8/21, 38%) using Google Glass to help individuals with various motor impairments provided them with an accessible interface to control an assistive robot [6] or an electric wheelchair [8]. This allowed them to operate a computer using only head or eye movements [7], facilitating everyday tasks with the use of voice commands and the touchpad [9], managing symptoms of Parkinson’s Disease (PD) [10-12], and providing speech feedback to patients with dysarthria to allow them to better adjust their volume [13]. Two studies (2/21, 10%) used Google Glass to help individuals with psychiatric or developmental disorders by recording blinking information as an indication of anxiety experienced by those with social anxiety disorder (SAD) [19] and by recording behaviors of individuals with autistic spectrum disorder (ASD) to provide better information to caregivers and clinicians [20]. Three studies (3/21, 14%) used Google Glass to assist in weight management by detecting and recording a person’s eating and drinking habits [21-23]. Two studies (2/21, 10%) provided individuals with real-time electrocardiograms (ECG) [25] or other physiological measurement feedback [24]. Finally, one study (1/21, 5%) allowed users to scan the ingredients of cosmetic products in drug stores to filter for common allergens [26].

**Table 2:** Summary of studies using Google Glass as clinician-focused interventions.

Source (country)	Health condition	Study design	Study setting	Google Glass (GG) use
Gillis et al, 2015 (United States)	Disaster relief	Pilot/feasibility study	Laboratory	Allows for audiovisual communication with each group of paramedics and the administrator; the virtual beacon component is used to eliminate the use for paper triage tags
Carenzo et al, 2014 (Italy)	Disaster relief	Pilot/feasibility study	Local – Field hospital	Scans triage tags to provide their information, timestamp, and Global Positioning System (GPS) coordinates and relay the information back to the hospital
Cicero et al, 2014 (United States)	Disaster relief	Pilot/feasibility study	Local – Airport	Facilitates communication with telemedicine physician disaster expert who can confirm the triage decision of the intervention team, and determines time of triage for each patient
Newaz and Eide, 2015 (Norway)	Disaster relief	Randomized control trial	Local – Neighborhood	Provides navigation and maps to first responders
Paxton et al, 2015 (United States)	Behavioral sciences	Randomized control trial	Laboratory	Uses PsyGlass app to facilitate the use of GG in behavioral, cognitive, and social research
Pappachan et al, 2014 (United States)	Diagnostics	Pilot/feasibility study	Hospital – Emergency department	Helps community health workers to identify certain disorders based on the patient demographics
Pascale et al, 2015 (Australia)	Nursing – Peripheral detection	Pilot/feasibility study	Laboratory	Improves detection in the periphery
Yuan et al, 2015 (China)	Neurology	Case study	Hospital – Neurology	Facilitates communication between physicians during a neurological examination
Chaballout et al, 2016 (United States)	Student training – health science students	Pilot/feasibility study	Classroom (university)	Presents a simulation in conjunction with real time performance of treatment on a manikin
Drake-Brockman et al, 2016 (China)	Anesthesiology	Pilot/feasibility study	Hospital – Anesthesiology	Uses heads-up display to facilitate monitoring patient vitals while performing procedures
Iversen et al, 2016 (United States)	Student training – physiotherapy students	Randomized control trial	Classroom (large, private, non-profit research university)	Captures 1st-person view of a procedure and displays it for learning purposes
Son et al, 2015 (United States)	Student training – otolaryngology residents	Pilot/feasibility study	Hospital - Otolaryngology	Uses the video capabilities to record resident encounters with patients
Spaedy et al, 2016 (United States)	Radiology	Quasi-experimental	Hospital – Radiology	Takes images of and displays X-rays for physician interpretation
Russel et al, 2014 (United States)	Student training – medical students (radiology)	Randomized controlled trial	Instructional testing room (University of Kentucky, School of Medicine)	Provides live instruction from an expert via Google Hangout
Wu et al, 2014 (United States)	Student training – medical students and radiology residents	Randomized controlled trial	Classroom (University of Arizona College of Medicine – Phoenix)	Facilitates procedures by showing real-time ultrasound images on the heads-up display
Widmer et al, 2014 (Switzerland)	Dermatology and Radiology	Exploratory study	Laboratory	Takes and analyzes images to facilitate interpretation and diagnostic decisions by presenting similar images to user
Stetler et al, 2015 (United States)	Cardiology	Quasi-experimental	Hospital – Cardiology	Captures images of the electrocardiogram (ECGs) and presents them on heads-up display to facilitate interpretation
Duong et al, 2015 (United States)	Cardiology	Exploratory study	Hospital – Cardiology	Records coronary angiograms were recorded to be reviewed on the heads-up display or transferred to a mobile phone
Jeroudi et al, 2014 (United States)	Cardiology	Exploratory study	Remote (location varied by physician reviewer)	Displays ECG images for interpretation
Vallurupalli et al, 2013 (United States)	Student training – medical students (cardiology)	Case study	Hospital – Cardiology (University of Arkansas for Medical Sciences, Division of Cardiology)	Facilitates collaboration between cardiology attending and resident in clinical training settings
Benninger, 2015 (United States)	Radiology	Pilot/feasibility study	Anatomy laboratory (Medical School)	Displays images captured by an ultrasound finger probe to teach medical students anatomy and simple interventions
Vaughn et al, 2016 (United States)	Student training – nursing students	Pilot/feasibility study	Classroom (Nursing School)	Presents a simulation in conjunction with real-time performance of treatment on a manikin
Zahl et al, 2016 (United States)	Student training – dental students	Exploratory study	Dental office	Records student SP station for later analysis
Feng et al, 2015 (United States)	Diagnostics – Human immunodeficiency virus (HIV) and cancer	Exploratory study	Laboratory	Takes rapid diagnostic tests (RDTs), images prostate specific antigen (PSA) tests, and images previously activated free PSA and total PSA RDTs
Spencer et al, 2014 (United States)	Pulmonology – airway assessment for burn victims	Case study	Hospital – Burn unit (Massachusetts General Hospital)	Facilitates assessment and management of the airway
Tully et al, 2015 (United States)	Student training – medical students (hospice)	Randomized controlled trial	University of Arizona, College of Medicine: Phoenix and local hospice organization	Records student standardized patient encounters for later analysis
Albrecht et al, 2014 (Germany)	Pathology – autopsy and postmortem examinations	Pilot/feasibility study	Hospital – Autopsy laboratory	Takes pictures of body for documentation during examination
Aldaz et al, 2015 (United States)	Chronic wounds	Pilot/feasibility study	Hospital – Wound care (Stanford Hospital and Clinics)	Uses SnapCap software to facilitate hands-free digital imaging and the tagging and transfer of images to patient’s electronic medical record in chronic wound care assessments
Chai et al, 2015 (United States)	Toxicology	Pilot/feasibility study	Hospital – Emergency department (urban academic hospital)	Sends photographs and videos to the toxicology supervisors; acts as a platform for instruction of 2nd-year medical staff
Chai et al, 2015 (United States)	Dermatology	Pilot/feasibility study	Hospital – Emergency department (urban academic hospital)	Allows teledermatolgosists to complete a dermatology assessment via live video feed after in-person consultation by a resident

**Table 3:** Summary of Google Glass approach as patient-centered interventions.

Source (health condition)	Purpose	Intervention description
Anam et al, 2014 (Ophthalmology – visual impairment)	To allow people with vision impairments gain the ability to determine non-verbal expressions	Expression is the type of feature addition that is being used
		It analyzes changes in facial expression and relays that information in the form of captured frames to user
		Helps user change their posture to better capture the facial expression
Garcia and Nahapetian, 2015 (Ophthalmology – visual impairment)	To help guide people with visual impairments navigate indoor environments	Extract floor regions from images captured from GG to help guide the individual
		An app is installed in GG that starts the camera and sends image frames to the mobile phone
		An app is also installed that analyzes the floor plans and then sends it to the mobile phone through Bluetooth
		Images that are captured contain the walls, floor, and ceiling
Pundlik et al, 2016 (Ophthalmology – visual impairment)	To use vision magnification to aid in the completion of tasks	Leverages zoom capabilities of GG
		Students are assigned tasks that involve the calculator and music player apps
		Performance on these tasks is measured
Hwang and Peli, 2016 (Ophthalmology – advanced age-related macular degeneration)	To augment the vision of the wearer so that they have improved vision	Vision enhancement tool is added to GG
		Participant wears GG which now warps the camera image to improve vision
		Images that the vision enhancement tool sees are then relayed to user in real-time
Tanuwidjaja et al, 2014 (Ophthalmology – colorblindness)	To help people with colorblindness see color	Alters the way people perceive color
		Applied Chroma, which is an app that detects color and relays that information to the participant
		Implemented the Ishihara test, which tests for color vision deficiency
		Implemented the Blackboard test that determines if a person can distinguish between green and orange
Lazewatsky et al, 2014 (Motor impairment)	To show that GG can be used in conjunction with the PR2 robot to recognize people and objects and then manipulate the space around it	GG Bridge Node receives sensor data from GG and transmits it to Robots and Systems software (ROS) messages and publishes a coordinate frame for GG
		ROS works with face detection; GG software also uses face detection and person recognition
Gips et al, 2015 (Motor impairment)	To help people operate a computer with only eye or head movements	Noggin software was developed to allow user to move a cursor across the screen through head movements
		Noggin displays yes, no, and enter on the screen
		Noggin uses the gyroscope to monitor head movements
		GG Gab, another software, allows user to spell out a message
Sinyukov et al, 2016 (Motor impairment – Locked-In Syndrome)	To help patients have better control over their wheelchairs	Patient uses the software installed on GG in conjunction with the motorized wheelchair
		GG monitors facial expressions of the patient
		GG’s audio monitoring is used to understand voice commands and then relay the instructions to the motorized wheelchair
Malu and Findlater, 2015 (Motor impairment – upper body)	To assess the accessibility of GG for individuals with upper body motor impairments	Using voice commands and the touchpad to go through day-to-day activities
		Touchpad on GG was on the right arm of the device and senses taps and swipes through voice commands
		Output is projected on the heads-up display
		Participants completed tasks using swipes and tasks function
		Participants then used a scale to rate the comport and ease of the touchpad and visual display
McNaney et al, 2014 (Motor impairment – Parkinson’s Disease [PD])	To help people with PD counteract their symptoms by allowing them to carry out the normal functions of a mobile phone using voice commands, cueing for freezing gait	GG was used to manage social cues and alert the user
		GG monitored movement and told the participant when they were freezing so that they could actively try to stop the behavior
McNaney et al, 2015 (Motor impairment – PD)	To help monitor speech loudness issues and provide feedback to help with self-management	Developed the LApp app that monitors loudness
		Participants used the app for a set amount of time while carrying out a series of social interactions
		Indicating when the volume was inappropriate so the user could adjust to hit the target loudness
Zhao et al, 2016 (Motor impairment – PD)	To provide visual and auditory cues to aid in the modulation of gait	GG was used to detect gait issues and improve them through cueing
		Audiovisual cues were used, including a metronome, flashing light, optic flow, and a control (no cue)
		Participants underwent a series of walking tasks and their gait was then analyzed for stability and freezing
Pervaiz and Patel, 2014 (Motor impairment – Dysarthria)	To help patients monitor their low volume in order to self-regulate and to provide clinicians with feedback to adjust therapy	Developed the SpeedOmeter software that compares vocal loudness to ambient noise
		Provides feedback to user on their volume
		System provides usage and performance history for user
		Notifies patient of their volume so they can adjust
Miranda et al, 2014 (Psychological/Developmental – SAD)	To assess the feasibility of using GG to monitor blinking rates in individuals with social anxiety disorder	Monitor blinking behaviors
		Used to gather data from the infrared (IR) sensor
		The app dealt with IR data gathering, data processing, and HTTP communication
		App processes the data and calculates when the user blinked
Voss et al, 2016 (Psychological/Developmental – ASD)	To monitor life activities and allow for analysis of autism behaviors	Participant uses GG to record everyday behaviors
		Caregiver reviews system highlights and emotional moments so they are easily accessible for the reviewer
		Caregivers can tag parts of the video that are especially important and add comments to the video
Mirtchouk et al, 2016 (Eating monitoring)	To accurately track an individual’s eating habits and provide feedback to help with self-regulation	GG sensor was used to detect head movement that was specific to eating
		Participants ate what they wanted and when they wanted and GG was supposed to detect when they were eating and for how long
		Participants were allowed to do other activities when eating their meals
Rahman et al, 2015 (Eating monitoring)	To detect a person’s eating and drinking habits	Records a person’s eating and drinking habits through head movements
		Helps people with obesity and diabetes
		Developed the Glass Eating and Motion (GLEAM) dataset
		Participants ate, walked, and did other activities during the monitoring period
		Participants did not interact with GG but simply wore it
		GG sensors recorded movement
Ye et al, 2015 (Eating monitoring)	To detail eating habits to help weight reduction	Collects images of the person’s day from their perspective every 30 seconds
		Amazon’s Mechanical Turk is a human computation platform that can determine eating behaviors and is used to identify when a person is eating
Hernandez et al, 2014 (Physiological measurements)	To measure heart rate and breaths per minute	Participant would wear GG, and GG’s accelerometer, gyroscope, and camera were used to find user’s pulse and respiratory rates
		The recording was done in several different positions including, sitting, standing, and lying down
Richer et al, 2015 (Physiological measurements)	To use the DailyHeart app to monitor ECGs	GG presents ECG signals to user in everyday life
		Signals are processed in real-time and classify the user’s heart beats
		It will store data in an internal database
Wiesner et al, 2015 (Allergies)	To give consumers information of possible allergens in cosmetic products	An app is developed for GG whose purpose is to scan products
		User scans the product in the store and the GG app identifies the product
		User has uploaded the information of their specific allergies and the app compares the ingredients to the user’s profile
		GG indicates whether the user should buy the product and why

### Description of Google Glass Use as Clinician-Centered Interventions

Table 4 summarizes the Google Glass approach as clinician-centered interventions. Four of the clinician-focused studies (4/30, 13%) used Google Glass to assist in disaster relief by providing first responders with maps and navigational assistance [30], maintaining audiovisual communication with groups of paramedics and administrators [27], scanning triage tags [28], and performing teleconsultations with physician experts to confirm triage decisions [29]. Two studies (2/30, 7%) used Google Glass to help community health workers make more efficient diagnoses [32] and by allowing clinicians to retrieve images of similar cases [42]. Another (1/30, 3%) provided nurses information about peripheral stimuli to help them more efficiently manage their clinical environment [33]. Two studies (2/30, 7%) used the teleconsultation capabilities of Google Glass to improve the accuracy of neurological [34] and emergency dermatology [56] examinations. Nine studies (9/30, 30%) used Google Glass in student training situations to provide first-person demonstrations of procedures [37], record students in simulated patient interactions [38,49,52], enhance simulated interactions by projecting videos of the scenarios into their visual field [35,48], provide students with live instruction from an expert [40,46,55], and teach anatomy by providing real-time ultrasound imaging [47]. One study (1/30, 3%) used Google Glass to provide patient monitoring data to assist anesthesiologists and minimize distractions during procedures [36]. Five studies (5/30, 17%) used Google Glass to capture images of X-rays [39,44] and ECGs [43,45] that physicians then interpreted for significant findings. One study (1/30, 3%) used Google Glass to minimize head movements during ultrasound-guided procedures by projecting the images onto Google Glass [41]. One study (1/30, 3%) used Google Glass to take and analyze rapid diagnostic tests (RDTs) [50]. One study (1/30, 3%) helped clinicians evaluate burn patients by assisting in airway assessment [51]. One study (1/30, 3%) assessed the potential uses of Google Glass in autopsy or forensics settings by specifically evaluating the quality of images taken by Google Glass for documentation [53]. One study (1/30, 3%) leveraged multiple functions of Google Glass to assist with the treatment of chronic wounds [54]. Finally, one study (1/30, 3%) developed an app for Google Glass to facilitate behavioral, cognitive, and social research [31].

**Table 4:** Summary of Google Glass approach as clinician-centered interventions.

Source (health condition)	Purpose	Intervention description
Gillis et al, 2015 (Disaster relief)	To provide a hands-free way for doctors to be updated on the status and needed-care levels of critical-care patients	Developed a mesh network that covered a set area to allow communication between users and the hospital
		Users wore GG and could communicate with each other across the lake
		Users were then able to use the information they were getting in the field, record it, and relay it back to the hospital
Carenzo et al, 2014 (Disaster relief)	To aid in nontechnical skills in the management of disasters and mass casualty incidents	Used an app to GG to guide a Simple Triage and Rapid Treatment Triage visually
		Focused heavily on casualty identification, therefore the facial recognition capabilities for GG were used
		Visual information was then relayed to a secondary location for others to monitor
Cicero et al, 2014 (Disaster relief)	To streamline the triage system and then also offer consultations from an expert physician to those onsite	Paramedics used GG to communicate with an offsite physician disaster expert
		They assigned triage levels to victims using the SMART Triage System
		Offsite physician had an audio-video interface with paramedics so they could be observed in the offsite location
Newaz and Eide, 2015 (Disaster relief)	To provide direction to first responders in a new area	One group used GG as a tool for navigation
		The other group used a different device to navigate an unfamiliar neighborhood
		The route was preset on GG or the other device
Paxton et al, 2015 (Behavioral sciences)	To determine how interpersonal dynamics in conversation are affected by the environment	The app PsyGlass was created for GG
		The students wore GG and were presented with a series of red or blue lights as well as audio stimuli
		They had a conversation with the experimenter and their head movements were recorded through the GG accelerometer
Pappachan et al, 2014 (Diagnostics)	To assist community health workers to more efficiently diagnose patients	Uses Rafiki, a GG software that calculates age and gender and other characteristics to diagnose a patient
		Correlates between diseases, symptoms, and patients to determine the problem
Pascale et al, 2015 (Nursing – peripheral detection)	To help clinicians, such as nurses, pay attention to multiple patients while away from their station	Provided stimuli in the periphery of the nurses
		GG was used to detect and notify the nurses when something was presented in their peripheral vision
Yuan et al, 2015 (Neurology)	To make a neurological examination as accurate as possible through collaboration	A woman that suffered a right-sided dysphagia and asthenia was in the emergency department with a suspected stroke
		A local physician lacking neurological knowledge used GG to establish a teleconsult with a remote specialist who guided the physician in evaluating the patient
Chaballout et al, 2016 (Student training – health science students)	To teach health care students to respond to respiratory distress	Students watched a video while wearing GG
		Video showed a patient in respiratory distress
		Students then performed a procedure to aid respiratory distress on a manikin in front of them
Drake-Brockman et al, 2016 (Anesthesiology)	To allow anesthesiologists to monitor vitals of patients during procedures	AnaeVis was developed to run on GG, which provides visualization of patient monitoring data
		Anesthetists wore the device while treating the patient and the signals were shown and recorded
Iversen et al, 2015 (Student training – physiotherapy students)	To record 1^st^-person view of procedures demonstrated by instructors to relay to students for training purposes	Faculty member wore GG during the performance of clinical skills
		Video of clinical skill performance was then shown to students for the purpose of teaching
Son et al, 2015 (Student training – otolaryngology residents)	To improve otolaryngology resident training by capturing 1^st^-person recordings of clinic encounters for later evaluation	Residents were recorded in an outpatient clinic by patients
		Patients were then given a survey to complete that rated their satisfaction level with their visit
		Video information was evaluated by two different parties and a review was given back to residents
Spaedy et al, 2016 (Radiology)	To improve the efficiency of remote chest X-ray interpretation	Fellows reviewed 12 chest X-rays with 23 major findings by viewing the image on GG, viewing an image taken by GG on a mobile device, and viewing the original X-ray on a desktop computer
		One point was given for each major finding
Russel et al, 2014 (Student training – medical students [radiology])	To determine if GG could provide telementoring instruction in bedside ultrasonography	Students wore GG and received real-time telementoring education
		Telementoring was done by an expert at a different location
		Students’ goal was to obtain best parasternal long axis cardiac imaging using a portable GE Vscan
Wu et al, 2014 (Student training – medical students and radiology residents)	To minimize the amount of distraction caused by monitors during ultrasounds	Medical practitioner wore the GG during the ultrasound procedure
		GG screen projected images and video to the wearer
		Practitioner’s hand movements and eye movement were recorded to see if there was improvement
Widmer et al, 2014 (Dermatology and Radiology)	To improve diagnostics in dermatology and cardiology	Participants would wear GG during a consultation
		ParaDISE app was developed to be a medical image retrieval system
		GG’s visual and photo taking capabilities were utilized and then the photograph was sent into the interface and could be matched with similar images
		Those similar images were then sent to the wearer
Stetler et al, 2015 (Cardiology)	To capture and facilitate the interpretation of ECGs	ECGs were selected that had important findings
		GG zoom capabilities were used to identify each finding
		Every time a participant identified a finding they received one point
		ECGs were captured using the video function of GG
Duong et al, 2015 (Cardiology)	To facilitate the interpretation of coronary angiograms	GG’s video function was used to record angiograms with specific findings
		Students were then told to try to determine each of the findings in the angiograms
Jeroudi et al, 2014 (Cardiology)	To facilitate the interpretation of ECGs	Physicians wore GG and looked at the ECG image on the screen
		Physicians wore GG and viewed a photograph of the ECG taken using GG and then viewed on a mobile device
		Results were then compared to other methods of viewing ECGs
Vallurupalli et al, 2013, (Student training – medical students [cardiology])	To improve resident training by streaming the view of residents during simulations to attending physicians for consultation	Residents wore GG while working through four scenarios in cardiovascular practice
		Live video of the scenarios taken by GG was streamed to a mobile phone or personal computer used by the attending physician
Benninger, 2015 (Radiology)	To facilitate teaching anatomy to medical students	Students familiarized themselves with GG for 10-30 minutes using a program called MiniGames
		Students were then given tutorials in groups of 3-5 while using GG with a finger probe to identify neuromuscular and organ structures and spaces in the limbs and cavities
		Students were tested during 7 separate laboratory examinations over 1 year to identify the same structures and practice procedures
Vaughn et al, 2016 (Student training – nursing students)	To increase the perception of realism in nursing student simulations	Students were allowed 10 minutes to familiarize themselves with GG before the intervention
		Students were then given the patient report and started the simulation in which GG projected a video of an acute asthma exacerbation scenario
		1-2 Certified Healthcare Simulation Experts evaluated students’ performance
Zahl et al, 2016 (Student training – dental students)	To facilitate self- and peer-assessment of standardized patient (SP) interactions for dental students	3rd-year dental students volunteered to record their SP encounter using GG while a traditional static camera simultaneously recorded
		All GG and static camera videos were later reviewed during Behavioral Patient Management small group discussions
		Students rated how effective each type of video was for assessing communication skills
Feng et al, 2015 (Diagnostics – HIV or cancer)	To improve the efficiency of immunochromatographic diagnostic test analysis	One or more RDTs, either HIV (qualitative) or PSA (quantitative), labeled with QR codes were imaged using GG
		Images were automatically transmitted to a digital server that located all RDTs and produced a quantitative diagnostic result, which was reported to user
Spencer et al, 2014 (Pulmonology – airway assessment for burn victims)	To facilitate airway assessment of burn patients requiring surgery	GG was worn by physicians during two cases of burn patients requiring airway assessment
		Documentation of procedure by GG was evaluated after the intervention
Tully et al, 2015 (Student training – medical students [hospice])	To facilitate medical student self-evaluation after end-of-life SP encounters	2nd-year medical students participated in end-of-life SP encounters where the SP was wearing GG to record the encounter
		Students then reviewed GG and traditional videos
Albrecht et al, 2014 (Pathology – autopsy and postmortem examinations)	To evaluate the feasibility of using GG in a forensics setting	Two physicians wore GG during 4 autopsy and postmortem examinations and took images using both GG and a traditional digital single lens reflex (DSLR) camera
		Six forensic examiners evaluated the images for quality
Aldaz et al, 2015 (Chronic wounds)	To facilitate photo documentation of chronic wounds for long-term care	Wound care nurses used SnapCap software on GG to take images, tag, and transfer them to patient electronic medical records
		Image quality and ease of use were evaluated
Chai et al, 2015 (Toxicology)	To facilitate toxicology teleconsultation in the emergency department	Emergency medicine residents wore GG while evaluating poisoned patients
		Real-time video of physician findings was transmitted to toxicology fellows and attendings for evaluation
Chai et al, 2014 (Dermatology)	To facilitate dermatology teleconsultation in the emergency department	Patients first had a standard dermatology consultation (phone call and sometimes a static photo of the rash) with a dermatology resident
		Patients were then evaluated by the dermatology chief resident through a real-time video filmed by the patient (wearing GG) and viewed by the physician on a tablet

### Feasibility and Acceptability of Google Glass as Patient-Centered Interventions

Table 5 summarizes the user satisfaction results of the patient-centered interventions (see Multimedia Appendix 3 for more technical results). Overall, participant feedback on the comfort and ease of use of Google Glass in patient-centered interventions was very positive. Of the participants with visual impairments, Anam et al reported a median usability score of 4.6/5 [14], and Tanuwidjaja reported that a majority of participants believed the Google Glass intervention would be useful in everyday life [18]. Among the participants with motor impairments, namely Parkinson’s Disease (PD), while overall reactions were positive [12] and some believed that Google Glass allowed them to do things they were not previously able to do independently [11], there were also some consistent frustrations expressed. For example, some experienced difficulties using the touchpad and voice navigation features as a result of tremors and dysarthria associated with the disease [10,11]. While participants with ASD reported positive experiences using Google Glass [20], participants using it to collect physiological data reported privacy concerns and found a smartwatch to have better usability [25]. Some of the common complaints reported were overheating of the device [14,15,20], its relatively short battery life [6,14,15,17,18,22,24], poor quality camera [17,18], perceived stigma when wearing the device in public [14,25], wireless connectivity issues [26], and concerns about privacy and the protection of confidential information [11,25]. These results suggest the potential for future research and implementation to support patients if Google can address some of the device’s technological limitations. However, the issues experienced by PD patients due to dysarthria and tremors should be addressed in interventions related to motor impairments.

**Table 5:** Feasibility and acceptability of Google Glass as patient-centered interventions.

Source (health condition)	User satisfaction results
Anam et al, 2014 (Ophthalmology – visual impairment)	Participants completed 5-point Likert scale on usability of the Expression system (a score of 5=the best): Learnability ‒ median 4.1, interquartile range (IQR) 0.7; Informativeness ‒ median 4.5, IQR 1.0; Usability ‒ median 4.6, IQR 0.7; User Satisfaction ‒ median 4.5, IQR 1.0; Willing to Use ‒ median 3.7, IQR 0.7. The relatively low score for “Willing to Use” can be attributed to perceived uncertainty in social acceptability of wearing a device such as GG.
Tanuwidjaja et al, 2014 (Ophthalmology – colorblindness)	4/6 participants reported they found Chroma system useful in performing study tasks and would find it useful in everyday life. Two participants expressed concerns about system lag time in switching between modes. One participant did not find the system helpful because his vision test scores worsened when using Chroma.
Malu and Findlater, 2015 (Motor impairment – upper body)	Participants rated system features on a 5-point scale (1=very easy/comfortable to 5=very difficult/uncomfortable): Visual Display ‒ comfort median 2, mean 2.2, SD 1.2; ease median 2, mean 2.2, SD 1.2; Touchpad Gestures ‒ comfort median 3, mean 3, SD 2.2; ease median 2, mean 2.7, SD 1.9; Voice Commands ‒ ease median 1, mean 1.7, SD 1.2. For the reciprocal tapping task, most (N=8) found the large touchpad easiest to use, and most (N=7) found the large touchpad to be most physically comfortable.
McNaney et al, 2014 (Motor impairment – PD)	Study exit interviews identified some concerns with usability of and patient satisfaction with GG. Some felt wearing GG in public drew unwanted attention, and 3/4 participants reported they would not wear GG in certain settings due to safety concerns. All participants experienced frustration when certain features, such as voice recognition and navigation, were difficult to use in everyday life or did not work. However, when the features were working properly, user satisfaction was high. GG enabled some to do things others without PD can do on mobile phones. Overall, reactions to GG were positive and showed appreciation for how GG could be used to help those with PD.
McNaney et al, 2015 (Motor impairment – PD)	Study exit interviews revealed mixed reactions to LApp program, with some finding significant improvement in and confidence with their speech volume and others reporting the program performance was inconsistent. Additional frustrations were related to GG’s short battery life and difficulties navigating the touchpad because of PD-related tremors.
Zhao et al, 2016 (Motor impairment – PD)	Most users found GG easy or very easy to use (N=7/11) and the instructions on screen clear or very clear to read (9/12). One user particularly liked the bone-conducting headphone because the metronome was less audible to others around. Some participants disliked GG’s placement of the visual display in the upper right corner (n=3) and suggested images be projected binocularly (n=1) or more focally (n=2) in the visual field. They suggested verbal instructions (n=9), rhythmic music (n=2), and postural feedback (n=1) as additional cues for the app and that cues be provided only when needed (n=2).
Voss et al, 2016 (Psychological/Developmental – ASD)	Review of videos of participants using GG system at home showed that children reported positive experiences with the activities at home and stated they viewed the system as a toy. However, the device heated up to uncomfortable levels if worn too long.
Richer et al, 2015 (Physiological measurements)	Participants completed a qualitative assessment of their experience using *DailyHeart* on GG. Mean usability rating for smartwatches (4.2) was higher than GG rating (2.8). Almost a third of participants were afraid that health data stored in Google Fit could be misused by third parties. In comparing the use of *DailyHeart* on GG and on Android Wear, Wear outperformed GG on all measures (appearance, features, handling, distraction, and overall usability).

### Feasibility and Acceptability of Google Glass as Clinician-Centered Interventions

Table 6 summarizes user satisfaction and technical results of the clinician-centered interventions (see Multimedia Appendix 4 for more technical results). The clinician-centered studies, which also varied greatly in specializations and uses of Google Glass, reported more inconsistent reactions regarding the utility of the device. Many of the studies reported technical frustrations similar to those mentioned in the patient-centered interventions—specifically, short battery life [29,31,35,37,41,42,48,50,53], device overheating [31,35,37,41], difficulties with wireless network connection [29,35,37,41,42,48,55], and privacy concerns [28,31]. Multiple studies reported that, while the device was generally comfortable to wear and did not distract from the clinician’s work, it did not significantly improve outcomes or the clinician’s efficiency [29,37,53]. The main sources of frustration specific to clinician use were the size and quality of images taken with and viewed through the device [42,45,50], difficulties in taking images and videos, and keeping patient monitors in the clinician’s line of vision due to the fact that the Google Glass follows a person’s head movements instead of gaze [33,36,49]. Specifically, Spaedy et al found that clinicians were dissatisfied viewing images of chest X-rays through Google Glass but were impressed with the images taken by the device and viewed on a mobile phone or computer [39]. In addition, Stetler et al, Duong et al, and Jeroudi et al reported that cardiologists were generally not confident with their interpretations of ECGs viewed through Google Glass [43-45]. However, other studies, in particular the ones that used Google Glass as a tool for training students, found that students had overall positive reactions to Google Glass and would recommend its future use [35,36,41,47-49,52]. For example, Chaballout et al found that most students (10/12, 83%) recommended its continued use in clinical simulations [35]. Similarly, Wu et al found that a majority (88%) of the medical students and radiology residents would be likely to use ultrasound visualization through Glass instead of a traditional monitor [41]. One exception to this trend was a study by Iversen et al, which reported that a majority of the physiotherapy students (26/39, 67%) found Google Glass’ video quality unacceptable, and many (23/39, 59%) did not feel the device enhanced their learning experience [37]. Despite this contradiction, these results suggest that the greatest potential for Google Glass implementation to support clinicians lies in student training.

**Table 6:** Feasibility and acceptability of Google Glass as clinician-centered interventions.

Source (health condition)	User satisfaction results
Cicero et al, 2014 (Disaster relief)	First responders using GG completed a survey assessment after the intervention, and their responses supported the idea that GG does not make a significant improvement in disaster triage.
Yuan et al, 2015 (Neurology)	Local physicians found that holding a mobile phone to provide the consulting specialist live images on GG was inconvenient.
	Teleneurohospitalists using GG did not feel the system allows for patient evaluation similar to what would be achieved in-person.
Chaballout et al, 2016 (Student training – health science students)	Participants were asked to complete 2 post-intervention surveys, a 13-item Student Satisfaction and Self-Confidence in Learning Scale and a 20-item Simulation Design Scale (scale for both measures was 1=strongly disagree to 5=strongly agree). Most students recommended continued use of GG in clinical simulations (N=10/12). They also reported high mean scores on the simulations’s design and satisfaction with the simulation to promote learning and self-confidence in learning.
	Simulation Design Scale (mean [SD]): Objectives and information ‒ 4.65 (0.18); Support ‒ 4.85 (0.04); Problem solving ‒ 4.53 (0.30); Feedback/guided reflection ‒ 4.85 (0.14); Fidelity (realism) ‒ 4.67 (0.12)
	Student Satisfaction and Self-Confidence with Learning (mean [SD]): Satisfaction with current learning ‒ 4.67 (0.13); Self-confidence in learning ‒ 4.35 (0.60).
Drake-Brockman et al, 2016 (Anesthesiology)	Anesthetists participating in the intervention were asked to complete a survey including a Likert scale and freeform questions: 78% would use GG again, 58% would recommend GG to colleagues, 21% felt GG improved patient management, 90% reported GG was comfortable to wear, 86% reported that information presented on GG was easy to read, 56% would wear GG in view of patients, 75% felt positive about using GG in the operating room environment, 82.5% reported that wearing GG did not distract from patient management.
Iversen et al, 2015 (Student training – physiotherapy students)	Students who used GG in the study answered questions about the technology after the intervention. 67% (26/39) of students evaluated GG video quality as not acceptable (score of ≤2 on the Likert scale), and 59% (23/39) of students reported using GG did not enhance their learning experience.
Spaedy et al, 2016 (Radiology)	Participants responded to a 5-point Likert scale about the quality of GG images and their confidence about their interpretation. When viewing images through GG, 87% (13/15) were dissatisfied with the image and unsure that such a small display would be able to provide the necessary level of detail. 80% (12/15) were impressed with image clarity taken via GG and viewed on the mobile device.
Wu et al, 2014 (Student training – medical students and radiology residents)	Participants who used GG responded to a post-exercise survey. 87% reported GG was comfortable to use for ultrasound guidance. 88% reported they would be likely to use ultrasound visualization through GG as opposed to traditional monitors (18% very likely, 35% moderately likely, 35% somewhat likely). 78% indicated they would “very likely” be interested in future research studies involving GG in medical simulation and education.
Stetler et al, 2015 (Cardiology)	Physicians responded to a 5-point user-experience Likert scale after the intervention. 58% (7/12) were satisfied with GG image quality of ECGs. 50% (6/12) were confident in their interpretation when using GG.
Duong et al, 2015 (Cardiology)	Participants responded to a post-study survey regarding their satisfaction with image quality and comfort making clinical recommendations. 10% (1/10) were “neutral” regarding quality and giving recommendations. 60% (6/10) of physicians were “somewhat satisfied” and would be “somewhat comfortable” giving recommendations. 30% (3/10) were “very satisfied” and would be “very comfortable” giving recommendations.
Jeroudi et al, 2014 (Cardiology)	Participants completed subjective ratings on a 5-point Likert scale regarding image quality and their confidence of ECG interpretation. 75% (9/12) were dissatisfied with the ECG image quality when viewing via GG. 83% (10/12) were not confident in their interpretation when viewing via GG. 58% (7/12) were neutral about ECG images taken by GG and viewed on mobile phones. 58% (7/12) were more confident in their interpretation when viewing the GG image on a mobile phone than when viewing via GG.
Benninger, 2015 (Radiology)	Participants responded to a 5-point Likert scale questionnaire. Did they enjoy the exposure to technology applying the triple feedback method? Average score 4.6. Would they prefer more time with the technology? Average score 4.8
Vaughn et al, 2016 (Student training – nursing students)	After the intervention, students responded to 2 surveys, the Simulation Design Scale and the Self-Confidence in Learning Scale (both 5-point scales from 1=strongly disagree to 5=strongly agree), to assess their perception of GG in the simulation: Independent problem-solving was facilitated, 4.75 (0.45); Resembled a real-life situation, 4.75 (0.45); Teaching methods were helpful and effective, 4.67 (0.65); Teaching materials were motivating and helpful, 4.58 (0.90); Confidence in mastering simulation content: 4.42 (0.51); Develops skills/knowledge applicable to a clinical setting, 4.83 (0.39)
Zahl et al, 2016 (Student training – dental students)	Students responded to 4 open- and closed-text items about using GG and static video for self- and peer-assessment. Students’ reported mean score was higher for GG recordings (84.61) than static video (79.74). Students reported that verbal communication was more easily assessed by reviewing GG video (23.87) than static video (22.17); paraverbal communication was more easily assessed by reviewing GG video (24.26) than static video (21.51); and nonverbal communication was more easily assessed by reviewing static video (19.78) than GG video (17.09).
Tully et al, 2015 (Student training – medical students [hospice])	Students responded to a 5-point Likert scale on how distracting they found GG during the intervention. 23% (7/30) reported a “positive, nondistracting experience.” 37% (11/30) reported a “positive, initially distracting experience.” 17% (5/30) reported a “neutral experience.” 10% (3/30) reported a “negative experience.” After reviewing the videos filmed with GG, 70% (16/30) believed that GG is worth including in the clinical skills training program.
Albrecht et al, 2014 (Pathology – autopsy and postmortem examinations)	Both participants agreed that GG was comfortable to wear but required more physical effort to capture images than a DSLR camera.
Chai et al, 2015 (Toxicology)	Study participants completed a survey immediately after the consult about their experience viewing a teleconsult through GG: 94% (17/18) were confident in the toxidrome after GG consultation as compared to 56% (10/18) who were confident after phone consultation.
Chai et al, 2014 (Dermatology)	All participants responded to a survey on acceptability of GG after their consultation. 93.5% (29/31) were overall satisfied with the video consultation. 22.6% (7/31) preferred care provided through mobile video communication technology over a standard face-to-face clinic visit. 74.2% (23/31) preferred care provided through mobile video communication technology over standard emergency department telephone consultation. 93.3% (28/31) would recommend the video consultation to others. 96.8% (28/30) felt comfortable that privacy was protected during the video encounter. 96.8% (30/31) were confident in the video equipment used.

## Discussion

### Principal Findings

In recent years, wearable devices such as wrist-worn accelerometers and head-mounted devices have become increasingly popular for their applications to everyday life as well as to various industries. While Google Glass, one of the more well-known head-mounted wearable devices, has yet to successfully break into the consumer market, various industries are eager to harness its potential in their fields. Medicine is one such industry; however, far greater attention has been paid to surgical applications than to nonsurgical ones. In this systematic review, we assessed existing evidence of the usability, benefits, and limitations of Google Glass to support both patients and clinicians in nonsurgical medical settings. Overall, the evidence was somewhat limited by a small number of studies fitting all inclusion criteria, small sample sizes, and other methodological considerations, particularly for statistical analysis. We included 51 studies that met our pre-set inclusion criteria, with the majority of studies describing clinician-centered interventions. There was a wide range of health conditions and uses of Google Glass. While information regarding age of participants was limited, the studies that did include age information were conducted with adults and none within pediatric populations. Many were conducted in laboratory, hospital, and student training settings, which indicates potential of university-affiliated teaching hospitals to integrate wearable technologies to make clinicians more efficient and provide clinical support to patients.

Unlike our systematic review, other recent reviews of the use of wearable technology in medicine included other heads-up devices besides Google Glass and did not distinguish between surgical and nonsurgical interventions [3,4]. Some of the uses of Google Glass in these studies include data visualization and video recording during surgery and interventional radiology, smart checklists, telementoring, virtual reality for education or pain management, interpretation of images, teleconsultation, teleconferencing, drug delivery tracking, patient empowerment, laboratory diagnostics, and forensic medicine [3,4].

A recent systematic review of medical applications of Google Glass in both surgical and nonsurgical settings found more globally positive support for the technology’s use in these settings [4]. However, this systematic review discussed a smaller sample of articles (n=21) that spanned surgical and nonsurgical medical interventions as well as scientific settings in general. Furthermore, original research studies on Google Glass in surgical interventions report fewer technical issues with the device and recommend strategies to overcome those that were encountered. One study, in which a pediatric surgeon wore Google Glass continuously for 4 weeks, reports with confidence that the photographic and video quality of the device was sufficient to capture all clinically relevant findings [57]. In contrast, many of the studies included in our analysis cited the photographic and video quality of Glass as a significant clinical limitation. While our findings regarding the limitations to the use of Glass, namely battery life, photographic and video quality, and streaming capabilities, were consistent with those encountered in surgical applications, the surgical studies seem to have made more progress in testing potential solutions. For example, one plastic surgeon used a USB-powered pocket battery to eliminate the need to recharge the device during the operation, noise-canceling headphones to enhance the sound transmitted by Glass, and a light emitting diode (LED) lamp headset to improve photo and video quality [4]. These findings support the potential of Google Glass to be even more beneficial in nonsurgical medical interventions if technical limitations are overcome either in newer models of the device or with the implementation of these solutions.

### Strengths and Limitations

Our systematic review has a number of strengths. First, our review was conducted following the recommendations and guidelines for rigorous systematic reviews methodology [58-60]. Second, we used a very sensitive search strategy guided by a librarian information specialist with no language restrictions to include as many relevant studies as possible and minimize possible publication bias. In addition, we searched other resources, including published systematic reviews, clinical trial registries, and different electronic databases. Finally, 2 authors completed the review process independently at all stages.

Our systematic review of the literature has some potential methodological limitations. First, similar to other systematic reviews, although our search criteria were comprehensive, we could have missed some relevant articles [61]. Second, we included only original research papers that have been published in peer-reviewed journals, and the possibility of publication bias with the tendency to report positive study results cannot be excluded [62]. Finally, a number of the studies included in our review had a relatively small sample size.

### Conclusions

Results regarding the feasibility, usability, and acceptability of Google Glass in nonsurgical medical settings were extremely varied, with more positive results being reported for patient-centered studies and student training settings. Further investigation with rigorous research designs evaluating the efficacy and cost-effectiveness of these more successful interventions in supporting patients and clinicians is warranted. These efforts would be beneficial in informing the base of evidence on the use of wearable devices, such as Google Glass, in medicine.

## Appendix

Multimedia Appendix 1 PRISMA checklist.

Multimedia Appendix 2 Search strategies.

Multimedia Appendix 3 Summary of the technical results of the patient-centered studies.

Multimedia Appendix 4 Summary of the technical results of the clinician-centered studies.

## Abbreviations

ASD: autism spectrum disorder
ECG: electrocardiogram
GG: Google Glass
PD: Parkinson’s Disease
RDT: rapid diagnostic test
SAD: social anxiety disorder
SP: standardized patient
