# Supplementary material for: Using Google Glass in Nonsurgical Medical Settings: Systematic Review
Source: JMIR Mhealth Uhealth. 2017 Oct 19;5(10):e159. doi: 10.2196/mhealth.8671 (PMC5668637; doi:10.2196/mhealth.8671)
Supplement: Multimedia Appendix 2 [file mhealth_v5i10e159_app2.pdf]

Google Glass search strings

PubMed, November 25, 2015

“Google glass”

= 80
Update Jan 4, 2017: 108 (39 new)

****

Embase, November 25, 2015

“Google glass”

= 94

Update Jan 4, 2017: 142 (48 new)

****

INSPEC, November 25, 2015

"google glass" AND (health* OR medic* OR surg*)
= 18

Update Jan 4, 2017: 27 (9 new)

****

CENTRAL, November 25, 2015

“google glass”
= 2

Update Jan 4, 2017: 8 (6 new)

****

IEEE Xplore, November 25, 2015
((("google glass"))) AND ((((health*) OR medic*) OR surg*))

=15

Update Jan 4, 2017: 22 (7 new)

******

Web of Science, November 25, 2015

“google glass”

=160

Update Jan 4, 2017: 294 (149 new)

****

Scopus, November 25, 2015

(TITLE-ABS-KEY ("google glass")) AND (medic* OR health* OR surg*)

=81

Update Jan 4, 2017: 226 (144 new)

November 2015: Total from all databases: 450

After de-duplication: 227 references

Added Compendex: 25 references

After de-duplication: 5 references

Jan 2017: Total new citations across all databases: 402

After de-duplication: 271 references
