# Supplementary material for: Using Google Glass in Nonsurgical Medical Settings: Systematic Review
Source: JMIR Mhealth Uhealth. 2017 Oct 19;5(10):e159. doi: 10.2196/mhealth.8671 (PMC5668637; doi:10.2196/mhealth.8671)
Supplement: Multimedia Appendix 3 [file mhealth_v5i10e159_app3.pdf]

Multimedia Appendix 3: Summary of the technical results of the patient-centered studies

**Source (Health Condition)**	**Technical Results**
Anam et al, 2014 (Ophthalmology – visual impairment)	Overall F-measure: 0.768<br><br>The high number of false positives can be attributed to overlapping expressions and involuntary movements.<br><br>Quantitative analysis of expression system: (precision, recall, F-measure):<br>• Smile: 0.913, 0.778, 0.840<br>• OpenSmile: 0.833, 0.833, 0.833<br>• Sleepy: 0.75, 0.692, 0.720<br>• Yawn: 0.625, 0.714, 0.667<br>• Looking up/down: 0.895, 0.708, 0.791<br>• Looking left/right: 0.933, 0.636, 0.756<br>• Average: 0.825, 0.727, 0.768
Garcia and Nahepetian, 2015 (Ophthalmology – visual impairment)	Number of correctly and incorrectly identified floor regions in the different sets of test images: (total images, % correctly identified)<br>• 1: 48, 75%<br>• 2: 20, 10%<br>• 3: 22, 81.82%<br>• 4: 27, 66.67%<br>• 5: 48, 77.08%<br>• 6: 24, 70.83%<br>• 7: 94, 65.96%<br>The floor detection does not work well on images taken when turning corners.
Pundlik et al, 2016 (Ophthalmology – visual impairment)	First set of experiments:<br>• Compression time reduced with increasing magnification for all 5 screen types.<br>• Reading time was usually longer than decoding time, especially at lower magnification.<br>• The calculator, map, and webpage required a longer reading time than the music player and host app.<br>• Decoding time was similar for all screen types.<br>• The performance of the reading operation was directly related to data size.<br><br>Second set of experiments:<br>• The time required to read and decode the screenshots on GG was similar for static and dynamic screens.
Hwang and Peli, 2016 (Ophthalmology – advanced age-related macular degeneration)	Measured contrast threshold (in log contrast sensitivity) without light diffuse filter: (with edge enhancement, without edge enhancement)<br>• Subject 1: 1.50, 1.50<br>• Subject 2: 1.50, 1.50<br>• Subject 3: 1.50, 1.50<br><br>Measured contrast threshold (in log contrast sensitivity) with light diffuse

	filter: (with edge enhancement, without edge enhancement)<br>• Subject 1: 1.50, 0.75<br>• Subject 2: 1.50, 0.75<br>• Subject 3: 1.50, 0.75
Tanuwidjaja et al, 2014 (Ophthalmology – colorblindness)	Results of each test:<br>• All participants had their online Ishihara color vision test results reduced from strong to mild.<br>• Five of the six improved considerably on the general pictures test.<br>• During the Blackboard test, Chroma helped the participants see the differences in color of chalk but not the color itself.<br>• Five of the six improved their scores on the general Clothes test.<br>• Two of four improved their scores on the Resistor test.<br>• Both participants who took the specialized Art test improved their scores to 100%.<br>• The one participant who took the specialized pH Strip test showed no improvement.<br>Overall, the general tests showed greater improvements than the specialized tests.
Lazewatsky et al, 2014 (Motor impairment)	Users were able to easily designate targets on a screen with GG, with 95% of interactions taking less than 6 seconds and the majority taking less than 3.
Gips et al, 2015 (Motor impairment)	Noggin provided proof of concept that the GG gyroscope could be used to sense head movements to move a mouse pointer with reasonable accuracy.<br><br>On Glass Gab, it took the participant 38 seconds to spell out the 14-character message.
Malu and Findlater, 2015 (Motor impairment – upper body)	Reciprocal tapping task: average tapping time per trial<br>• Small touchpads: 2.7s (SD = 1.3)<br>• Medium touchpads: 1.8s (SD = 1.0)<br>• Large touchpads: 2.0s (SD = 1.1)<br>A one-way repeated measures ANOVA showed a main effect of touchpad size on average trial completion time ($F_{2,18} = 8.57$, $p = 0.002$, $\eta^2 = 0.49$).<br><br>Location customization and tapping task: average trial times<br>• Small touchpads: 3.2s (SD = 1.5)<br>• Medium touchpads: 2.5s (SD = 1.3)<br>• Large touchpads: 2.2s (SD = 0.96)<br>A one-way repeated measures ANOVA showed a significant impact of touchpad size on tapping speed ($F_{2,18} = 9.55$, $p = 0.001$, $\eta^2 = 0.51$).
McNaney et al, 2015 (Motor impairment – Parkinson's Disease)	Averages of usage data from 3 days of LApp trial: participant (target TdB): times above target, times below target<br>• Susan (45): 8, 4<br>• Neil (45): 5, 12<br>• Jill (51): 9, 11<br>• Michael (48): 13, 2<br>• Jerry (55): 28, 5<br>• Robert (52): 32, 6

Zhao et al, 2016<br>(Motor impairment – Parkinson's Disease)	Freezing of gait:<br>• The number of FOG episodes per trial ($\chi^2(3) = 7.29$, $p = 0.063$) and the FOG duration ($\chi^2(3) = 2.42$, $p = 0.50$) were not significantly different among cueing conditions<br>• During 360° turns, fewer participants experienced FOG while using a cue and significantly less FOG episodes occurred per trial while using the metronome compared to no cues ($p < 0.05$, $z = -2.13$)<br><br>Stride length:<br>• All cues showed a significant decrease in stride length variability in comparison to no cues (metronome: -2.23 ± 0.56 cm (-7.1%), $t(335) = -3.97$, $p < 0.001$; optic flow: -1.84 ± 0.56 cm (-5.9%), $t(335) = -3.30$, $p < 0.01$)<br>• The metronome was associated with a significant increase in the stride length (2.22 ± 0.93 cm (2.6%), $t(334) = 2.38$, $p < 0.05$)<br>• The optic flow and LED were associated with a significant decrease in the stride length (optic flow: -2.44 ± 0.92 cm (-2.8%), $t(334) = -2.64$, $p < 0.01$; LED: -3.22 ± 0.93 cm (-3.8%), $t(334) = -3.45$, $p < 0.005$)<br><br>Walking speed:<br>• The metronome was associated with a significant increase in walking speed only during the doorway course (5.62 ± 0.22 cm/s (11.2%), $z = 2.51$, $p < 0.05$)<br>• The optic flow was associated with a significant decrease in walking speed during the wide and narrow turn courses (wide: -7.33 ± 0.22 cm/s (-7.5%), $z = -3.34$, $p<0.01$; narrow: -9.72 ± 0.22 cm/s (-10.7%), $z = -4.42$, $p < 0.001$)<br>• The LED was also associated with a significant decrease in walking speed during the wide and narrow turn courses (wide: -11.92 ± 0.22 cm/s (-12.1%), $z = -5.321$, $p < 0.001$; narrow: -13.1 ± 0.23 cm/s (-14.4%), $z = -5.79$, $p < 0.001$)<br><br>Cadence:<br>• All cues showed significant decreases in cadence for the narrow turn courses (metronome: -5.20 ± 1.75 steps/min (-4.6%), $z = -2.96$, $p < 0.01$; optic flow: -8.25 ± 2.00 steps/min (-7.3%), $z = -4.12$, $p < 0.001$; LED: -12.03 ± 2.35 steps/min (-10.7%), $z = - 5.13$, $p < 0.001$) and full turn courses (metronome: -4.35 ± 1.47 steps/min (-3.7%), $z = -2.97$, $p < 0.01$; optic flow: -5.48 ± 1.78 steps/min (-4.7%), $z = -3.08$, $p < 0.01$; LED: -5.17 ± 2.14 steps/min (-4.4%), $z= -2.41$, $p < 0.05$)<br>• The LED was associated with a significant decrease in cadence during the wide turn course (-8.02 ± 2.33 steps/min (-7.4%), $z = -3.45$, $p < 0.01$)
Pervaiz and Patel, 2014<br>(Motor impairment – Dysarthria)	The average loudness change of 4.52 dB in feedback condition is comparable to the average 4.68 dB achieved using conventional clinician-based intervention. Participants with loudness under 70 dB in no-feedback condition increased their loudness by more than 7 dB in feedback condition.

Miranda et al, 2014 (Psychological/Developmental – Social Anxiety Disorder (SAD))	One-tailed T-test over the average values of SBR and HR data before and after questions:<br>• The SBR (p > 0.5) and the HR (p < 0.01 but with a negative SD) of the subject with SAD did not rise significantly after an “aggressive” question<br><br>There was a significant difference in HR values while testing subjects of the SAD group in the light and aggressive questions (p < 0.05 for both), while subjects with no SAD showed no statistical difference (p > 0.05)
Voss et al, 2016 (Psychological/Developmental – Autism Spectrum Disorder (ASD))	Behavioral findings:<br>• Parents commented on improvements in child eye contact after using the system<br>Feedback mechanism findings:<br>• The optimal feedback mechanism is a combination of visual and audio feedback<br>Indicator findings:<br>• Children displayed a strong preference for the box indicator, were accepting of the line indicator, and did not like the triangle indicator
Kleinberg et al, 2016 (Eating monitoring)	Accuracy of food type classification for different combinations of motion and audio sensors:<br>• Acoustic, GG, right watch, and left watch: 82.7%<br>• Acoustic, GG, and right watch: 81.2%<br>• Acoustic, GG, and left watch: 78.5%<br>• GG, right watch, and left watch: 76.2%<br>• Acoustic, right watch, and left watch: 79.3%<br>• Acoustic and GG: 76.4%<br>• Acoustic and right watch: 77.7%<br>• Acoustic and left watch: 77.0%<br>• Right watch and left watch: 72.4%<br>• Acoustic: 67.8%<br>• GG: 62.3%<br>• Right watch: 66.0%<br>• Left watch: 61.4%
Rahman et al 2015 (Eating monitoring)	Experimental results:<br>• No eating was detected in 9 participants (2 frequently adjusted their prescription glasses, 1 moved abruptly in a rolling chair, 1 ate a very small meal)<br>• Shorter meals (less than 15 minutes) were less likely to be recognized<br>• 100% precision was achieved for 11 participants
Ye et al, 2015 (Eating monitoring)	Eating detection with Pebble Watch:<br>• Across 20 meals, subjects had a range of 28 to 52 HtM gestures for each meal (38 on average)<br>• The mean in-person precision was 94.48% with recall of 95.02%<br>• The mean cross-person precision was 92.8% with recall of 90.3%<br><br>Eating detection with combination of GG and Pebble Watch:<br>• SVM algorithm had an average cross-person accuracy of 97%, which is better than the accuracies of GG or Pebble Watch alone

Richer et al, 2015 (Physiological measurements)	Results of sensor evaluation: MAE (bpm) ± SD (bpm), PC<br>• Subject 1: 2.43 ± 2.91, 0.957<br>• Subject 2: 6.54 ± 6.58, 0.920<br>• Subject 3: 3.85 ± 3.45, 0.912<br>• Subject 4: 5.64 ± 4.29, 0.913<br>• Subject 5: 5.68 ± 5.05, 0.906<br>• Mean: 4.83 ± 4.46, 0.922
Wiesner et al, 2015 (Allergies)	Ambient light can negatively impact processing time, but the percentage of products identified correctly was much higher in ambient light in a real-life shopping environment at local drugstores. There were no major communication interruptions.

App: application; GG: Google Glass; SD: standard deviation; FOG: freezing of gait; SAD: social anxiety disorder; SBR: spontaneous blink rate; HR: heart rate; HtM: hand-to-mouth; SVM: support vector machine; MAE: mean absolute error; PC: Pearson correlation
