# Supplementary material for: Using Google Glass in Nonsurgical Medical Settings: Systematic Review
Source: JMIR Mhealth Uhealth. 2017 Oct 19;5(10):e159. doi: 10.2196/mhealth.8671 (PMC5668637; doi:10.2196/mhealth.8671)
Supplement: Multimedia Appendix 4 [file mhealth_v5i10e159_app4.pdf]

Multimedia Appendix 4: Summary of the technical results of the clinician-centered studies

**Source (Health Condition)**	**Technical Results**
Gillis et al, 2015 (Disaster relief)	Heat experiments:<br>• For less computationally intensive applications, GG stabilizes around 95°F, but increases when running a video application<br>• When running non-display background tasks (i.e. Wi-Fi connectivity) the heat stabilizes to around 73°F<br><br>Range experiments:<br>• Quality decreases while lag time increases with distance, but at large distances the quality is unusable and the device communication gets disconnected<br><br>Average ease of setup in lake simulation: average score (1 = low to 5 = high)<br>• Training on setup and use: 4.67<br>• Assembly: 4.00<br>• Equipment mobility: 4.00<br><br>Average ease of use in lake simulation: average score (1 = low to 5 = high)<br>• Video: 5.00<br>• Audio: 2.67<br>• Improved ability to serve purpose: 3.67<br>• Equipment mobility: 4.00<br>• Device and application: 4.00<br>• Application stability: 3.33
Cicero et al, 2014 (Disaster relief)	There was no significant difference in the triage accuracy between the intervention and control groups:<br>• 100% of patients mistriaged by the intervention group were undertriaged, while 28.6% of the patients mistriaged for the control group were overtriaged<br><br>The two patients triaged by the intervention group with help from the telemedicine physician took significantly more time to triage.
Newaz and Eide, 2015 (Disaster relief)	Time taken to navigate the route for participants using GG:<br>• G1: 26.23 s<br>• G2: 22.03 s<br>• G3: 24.37 s<br>• G4: 23.44 s<br>• G5: 24.39 s<br>• G6: 25.09 s<br>Time taken to navigate the route for participants using mobile phones:<br>• M1: 22.54 s<br>• M2: 25.50 s<br>• M3: 26.22 s<br>• M4: 23.27 s<br>• M5: 24.56 s

	• M6: 26.02 s<br><br>One of six participants using GG as well as one of six using mobile phones appeared frustrated, while the rest were categorized as confident.
Paxton et al, 2015 (Behavioral sciences)	Increases in lag significantly predicted decreases in r, the measure of interpersonal synchrony or convergence ($\beta = -0.50$, $p < 0.0001$):<br>• Partners' head movements were most strongly correlated at lag 0, or in moment-to-moment comparisons ($\beta = 0.19$, $p > 0.30$)<br><br>There was no significant difference between the noise and dual-task conditions and no significant effect of the interaction term ($\beta = -0.03$, $p > 0.60$).
Pascale et al, 2015 (Nursing – peripheral detection)	Comparing monitor and GG:<br>• Using GG, but leaving all other variables constant, significantly reduced the odds of detecting the dark gray tilted stimuli ($p < 0.001$)<br>• Odds of detection also dropped significantly ($p < 0.001$) when stimuli were presented at the far eccentricity compared to near eccentricity<br>• Presenting peripheral stimuli on GG at the far eccentricity dropped the odds of detection further ($p < 0.001$)
Yuan et al, 2015 (Neurology)	The teleneurohospitalist was able to quickly guide the local physician through a thorough evaluation and recommend the use of IV thrombolysis. The dysphasia resolved, the myodynamia of the right upper and lower limbs recovered from grade III to IV, and neurological function completely recovered, although an infarction spot was noted on CT scans.
Iversen et al, 2015 (Student training – physiotherapy students)	All students successfully completed the 160-minute session of vestibular dysfunction assessment and treatment skills:<br>• Students in the GG group scored better on the clinical skills check, with a median score of 19 (range 16-20) versus 18 (range 16-20; $p = 0.03$)<br>• There was no statistical difference between groups except in the category assessing execution and handling of patients during clinical assessment, in which 77.0% of the GG group and 59% of the control group performed satisfactorily ($p = 0.049$)<br>• 37% of the GG group and 31% of the control group performed the execution and handling of the treatment manoeuver satisfactorily in every section of the test ($p = 0.58$)
Son et al, 2015 (Student training – otolaryngology residents)	Patient survey:<br>• The scores of questions regarding the subject of better explanations ($p = 0.061$), listening carefully ($p = 0.123$), addressing patient questions ($p = 0.086$), spending adequate time ($p = 0.2$), and overall performance ($p = 0.0715$) decreased with the use of GG but none reached significance<br>• The score for displaying respect decreased significantly ($p = 0.0065$) with the use of GG<br><br>Faculty survey:<br>• The scores of questions regarding making eye contact ($p < 0.001$), asking open-ended questions ($p = 0.0015$), sitting down ($p = 0.0095$), better explanations ($p < 0.001$), listening carefully ($p < 0.001$), addressing patient questions ($p < 0.001$), displaying respect ($p < 0.001$), and spending adequate time ($p = 0.0005$) significantly improved with use of

	GG<br>• The scores for engaging with proper empathy (p = 0.0005) and overall performance (p = 0.014) significantly improved with the use of GG<br>• The score for proper instruction decreased with the use of GG but not to significance (p = 0.06)<br>• The score for handwashing increased with the use of GG but not to significance (p = 0.062)
Spaedy et al, 2016 (Radiology)	Average chest X-ray reading score (max. 23):<br>• Viewed through GG: 14.1 ± 2.2<br>• GG photo on mobile device: 18.5 ± 1.5<br>• Original X-ray on desktop computer: 21.3 ± 1.7<br>(p < 0.0001 between GG and mobile device, p < 0.0001 between GG and desktop computer, and p = 0.0004 between mobile device and desktop computer)
Russel et al, 2014 (Student training – medical students (radiology))	Both telementored students and live instruction students were able to obtain adequate images for EPSS measurement in each instance:<br>• Telementored students had a median image quality rating of 7.5 out of 10 (IQR = 6 to 10)<br>• Live instruction students had a median image quality rating of 8 out of 10 (IQR = 7 to 9)<br>• Only one of the six subjects who received no instruction was able obtain an adequate image, and median quality ratings for this group were 0 out of 10 (IQR = 0 to 2)<br>• There was no significant difference in the achievement of adequate images for EPSS measurement, nor in image quality between telementored and live instruction groups
Wu et al, 2014 (Student training – medical students and radiology residents)	Central line performance metrics:<br>• The GG on average took longer to perform the procedure at every training level, but only reached significance for the M4 (197 s vs. 91 s, p ≤ 0.05) and PGY3 (151 s vs. 52 s, p ≤ 0.05) students<br>• In the PGY3 training level, participants spent significantly more time focusing their gaze on the patients (48 s vs. 23s, p ≤ 0.05), as well as on the GG monitor (103 s vs. 29 s, p ≤ 0.05) than the non-GG group<br>• MS1 students wearing GG spent significantly more time (139 s vs. 47 s, p ≤ 0.05) looking at the monitor than the non-GG group<br>• Both groups at all training levels had similar experiences (p > 0.05) performing central venous lines on both live and simulated patients
Widmer et al, 2014 (Dermatology and Radiology)	Accuracy when searching for skin of forearm image:<br>• Without a keyword, 3 of 10 images were accurate<br>• With a keyword, 7 of 10 images were accurate<br><br>Accuracy when searching for lung CT image: number of accurate results<br>• Original photo (no keyword): 2/10<br>• Original photo (keywords lung+CT): 10/10<br>• Printed photo (no keyword): 0/10<br>• Printed photo (keywords lung+CT): 10/10<br>• LCD screen photo (no keyword): 1/10<br>• LCD screen photo (keywords lung+CT): 7/10

	Accuracy when searching for brain MRI image: number of accurate results<br>• Original photo (no keyword): 2/10<br>• Original photo (keywords brain+MRI): 8/10<br>• Printed photo (no keyword): 1/10<br>• Printed photo (keywords brain+MRI): 7/10<br>• LCD screen photo (no keyword): 1/10<br>• LCD screen photo (keywords brain+MRI): 6/10
Stetler et al, 2015 (Cardiology)	Average ECG reading score (max. 21):<br>• Viewed through GG: 12.4 ± 2.7<br>• GG photo on mobile device: 13.4 ± 4.4<br>• Original ECG on paper: 14.8 ± 4.4<br>($p = 0.45$ between GG and mobile device, $p = 0.15$ between GG and paper, and $p = 0.38$ between mobile device and paper)
Duong et al, 2015 (Cardiology)	Average coronary angiogram reading score (max. 17):<br>• GG recording viewed on iPad: 14.9 ± 1.1<br>• GG recording viewed on desktop computer: 15.2 ± 1.8<br>• Original angiogram: 15.9 ± 1.1<br>($p = 0.06$ between GG recording on iPad and original, $p = 0.51$ between GG recording on iPad and desktop, and $p = 0.43$ between GG recording on desktop and original)
Jeroudi et al, 2014 (Cardiology)	Average ECG reading score (max. 21):<br>• Viewed through GG: 13.5 ± 1.8<br>• GG photo on mobile device: 16.1 ± 2.6<br>• Original ECG on paper: 18.3 ± 1.7<br>• High-resolution camera photo on mobile device: 18.6 ± 1.5<br>($p = 0.0005$ between GG and GG image on mobile device, $p = 0.0005$ between GG and paper, and $p = 0.002$ between GG image on mobile device and paper, $p = 0.5625$ between camera image on mobile device and paper, $p = 0.125$ between camera image on mobile device and GG image on mobile device)
Vallurupalli et al, 2013, (Student training – medical students (cardiology))	Scenario 1: the trainee wearing GG studied and interpreted the ECG with the senior fellow, subtle signs of ischemia were discussed, and appropriate treatment was initiated.<br><br>Scenario 2: the cardiology fellow wore GG to contact his senior fellow who reviewed the ECG images in question, confirmed that the patient was in cardiac tamponade, and initiated appropriate treatment.<br><br>Scenario 3: the first-year cardiology fellow wore GG to perform an ICD interrogation with the guidance of a senior fellow, a correct diagnosis of ventricular tachycardia was made, and the patient was admitted for appropriate treatment.<br><br>Scenario 4: the cardiology fellow wore GG while performing a subclavian vein cannulation with the attending sitting outside the room and watching the first-person video, allowing the fellow to gain confidence on his own and the attending to assess the fellow.
Benninger, 2015 (Radiology)	Class success range (ability to wear GG while using SonicEye ultrasound finger probe to identify a structure/space during a 1 minute laboratory examination:

	81%-97%<br>• The initial lab examination score was 81% but improved across six examinations to 97%
Zahl et al, 2016 (Student training – dental students)	Overall performance:<br>• There was not a statistically significant difference between mean scores of GG and static camera videos on the VRAES ($t_{22} = 1.702$, $p = 0.103$)<br>• Students' reported mean score on the VRAES was higher for the GG recordings ($\acute{x}$ = 84.61) compared to the static camera recordings ($\acute{x}$ = 79.74)<br><br>Verbal communication:<br>• Students reported that verbal communication was more easily assessed using GG video ($\acute{x}$ = 23.87) than static camera video ($\acute{x}$ = 22.17) ($p = 0.048$)<br>• Students rated the GG video higher for every item on the verbal communication subscale<br><br>Paraverbal communication:<br>• Students reported that paraverbal communication was more easily assessed using GG video ($\acute{x}$ = 24.26) than static camera video ($\acute{x}$ = 21.51) ($p = 0.003$)<br>• Every item on the paraverbal subscale except for 'voice inflection' (95.7%) was rated by every student (100%) as easily or very easily assessed using the GG video<br><br>Non-verbal communication:<br>• There was a statistically significant difference in mean scores between the GG and static camera videos on the non-verbal communication subscale ($p = 0.044$), with students reporting that it was easier to assess non-verbal communication using the static camera video ($\acute{x}$ = 19.78) than GG ($\acute{x}$ = 17.09)<br>• A higher percentage of students rated the static camera video as more effective for self-/peer-assessment of non-verbal communication on every subscale item except 'eye contact with the patient'
Feng et al, 2015 (Diagnostics – HIV or cancer)	Qualitative decision evaluation using HIV RDTs:<br>• The validation achieved correct diagnosis on all 281 images of OraQuick ADVANCE Rapid HIV-1/2 antibody tests for up to even 5x dilution factors<br>• A second validation of 145 images was done, again achieving 100% accuracy<br><br>Quantitative decision evaluation using PSA RDTs:<br>• There was a linear relationship between PSA concentration (ng/mL) and the test line intensity values read for both free and total PSA<br>• In the free PSA experiments, concentrations above 20 ng/mL start saturating and break the linearity of the response curves<br>• Additionally, for 25 images GG was used to detect and evaluate all PSA tests with minimal differences in test line intensity compared to single RDT images

Spencer et al, 2014 (Pulmonology – airway assessment for burn victims)	In both cases, GG was used to document anesthetic airway management in real time, with minimal disruption to clinical care, under standard operating room settings.
Tully et al, 2015 (Student training – medical students (hospice))	GG was successfully used to record 30 student/SP encounters:<br>• There was one instance of hardware malfunction, but no other technical issues<br>• Each 9-12-minute session used about 25% of the GG battery life<br>• The built-in microphone did an excellent job picking up the voice of the SPs but was less successful picking up the lower-volume voice of the students
Albrecht et al, 2014 (Pathology – autopsy and postmortem examinations)	Average scores of Likert evaluation (1 = low to 5 = high) by 6 raters for images (102 DSLR, 192 GG) taken during autopsy examinations:<br>• Region of interest: 4.45 (DSLR), 3.81 (GG) ($p < 0.001$)<br>• Sharpness: 4.59 (DSLR), 3.58 (GG) ($p < 0.001$)<br>• Color: 4.25 (DSLR), 3.80 (GG) ($p < 0.001$)<br>• Brightness: 3.98 (DSLR), 3.64 (GG) ($p = 0.001$)<br><br>Average scores of Likert evaluation (1 = low to 5 = high) by 6 raters for images (102 DSLR, 192 GG) taken during postmortem examinations:<br>• Region of interest: 4.37 (DSLR), 3.77 (GG) ($p < 0.001$)<br>• Sharpness: 4.25 (DSLR), 4.22 (GG) ($p = 0.56$)<br>• Color: 4.20 (DSLR), 4.16 (GG) ($p = 0.59$)<br>• Brightness: 4.13 (DSLR), 4.02 (GG) ($p = 0.01$)
Aldaz et al, 2015 (Chronic Wounds)	Barcode scanning for patient identification:<br>• There was a statistically significant preference for hands-free barcode scanning for routine clinical care ($Z(15) = -3.873$, $p < 0.001$, $r = 0.71$)<br>• All nurses successfully used the SnapCap Glassware to read the patient barcode within four seconds<br><br>Voice-based documentation through video:<br>• Voice-based documentation through a brief video was strongly preferred by nurses ($Z(15) = -2.84$, $p = 0.005$, $r = 0.52$)<br>• Only 12.5% (N = 2/16) of nurses succeeded in using voice commands to launch the video recording feature on their first try<br><br>Double blinking to take photographs:<br>• There was a statistically significant preference for this feature ($Z(15) = -3.606$, $p < 0.001$, $r = 0.71$)<br>• One nurse accidentally took extra photos because GG registered her natural blinking as double blinking<br><br>Head tilt for zooming:<br>• This was the least preferred hands-free interaction method ($Z(10) = -1.897$, $p = 0.058$)<br><br>Speech-to-text annotation:<br>• 87.5% (N = 14/16) of nurses successfully saved the two wound

	annotations in four attempts or less<br>• As a result of poor network connectivity, 12.5% (N = 2/16) of nurses required more than ten attempts to successfully save the two wound annotations
Chai et al, 2015 (Toxicology)	18 of 19 attempted GG consults were successfully completed:<br>• 89% (N = 16/18) of consults were considered successful by the supervisory consultant<br>• Interruptions in the video feed occurred during four consults<br>• Interruptions in the audio feed occurred during one consult but did not affect the quality of the consult<br>• One consult had interruptions of video and audio feed, thus the consult was unusable<br>• The confidence of the supervisory consultants in diagnosing a specific toxidrome in the poisoned patient increased from 59% (N = 10) to 94% (N = 17) after a virtual exam using GG
Chai et al, 2014 (Dermatology)	91% (N = 31/34) attempted connections were completed and considered successful.

GG: Google Glass; CT: computerized tomography; IQR: interquartile range; LCD: liquid crystal display; MRI: magnetic resonance imaging; ECG: electrocardiogram; ICD: implantable cardiac defibrillator; HIV: human immunodeficiency virus; RDT: rapid diagnostic test; PSA: prostate specific antigen; SP: simulated patient; DSLR: digital single-lens reflex
